# Final Year Students' Knowledge on Basic Manual Wheelchair Provision: The State of Occupational Therapy Programs in Colombia

**DOI:** 10.1155/2020/3025456

**Published:** 2020-04-27

**Authors:** María L. Toro-Hernández, Liliana Alvarez, María C. Vargas-Chaparro, Mary Goldberg

**Affiliations:** ^1^Department of Physical Therapy, Universidad CES, Medellin, Colombia; ^2^School of Occupational Therapy, Western University, London, Canada; ^3^School of Occupational Therapy, Escuela Colombiana de Rehabilitación, Bogota, Colombia; ^4^International Society of Wheelchair Professionals, Pittsburgh, PA, USA; ^5^Human Engineering Research Laboratories, University of Pittsburgh, Pittsburgh, PA, USA; ^6^Department of Rehabilitation Science and Technology, University of Pittsburgh, Pittsburgh, PA, USA

## Abstract

Access to personal mobility is a human right and as such, it implies the provision of wheelchair services for those with mobility impairments that need one. Lack of appropriately trained personnel is a major contributor to the gap in access to wheelchairs. Assistive technology provision is one of the core competencies of occupational therapists. The goal of this study was to assess the current wheelchair provision knowledge of final year occupational therapy students in Colombia as measured by the International Society of Wheelchair Professionals Basic Wheelchair Service Knowledge Test. A total of 83 students from 7 universities took the test. None of the students met the 70% passing threshold. The highest scores were in the assessment domain while the lowest in the fitting and user training domains. These results suggest that the current wheelchair provision education received in these programs do not meet the World Health Organization guidelines on appropriate wheelchair provision. The implementation of strategies to improve current wheelchair provision education in Colombian occupational therapy programs is granted.

## 1. Introduction

The World Health Organization (WHO) estimates that 1% of the world's population needs a wheelchair as their primary means of mobility [1]. Twenty million people do not have access to an appropriate wheelchair [2]. In other words, only around 30% of those who need an appropriate wheelchair have access to one, and this percentage decreases in low- and middle-income countries (LMIC) [2]. This situation poses a major threat to sustainable development, as access to personal mobility is a human right and it is necessary for people with disabilities to access other rights such as education, employment, and independent living [3, 4].

An appropriate wheelchair is one that meets the user's needs as well as the environmental characteristics, is affordable, of good quality, and is available and maintainable locally [1]. Limited access to appropriate wheelchairs is a multifactor issue [5]. Poor understanding of the needs often results in a lack of policies that promote and finance appropriate products [5]. In addition, limitations in the availability and quality of provision of services that can be delivered by trained personnel further restrict access to assistive technology [4, 6, 7]. Even in situations where planned services to increase access exist, a shortage of skilled personnel can result in a significant barrier to the implementation of such services [6, 8]. In order to address this challenge, local higher education institutions are called upon to educate and equip rehabilitation professionals, among others, with the tools to meet the provision needs for assistive technology, including wheelchairs [6]. Supporting competency development of providers is cited as one of the top ten priority actions from a recent international “Wheelchair Stakeholders Meeting” supported by USAID, World Learning [9], and the International Society of Wheelchair Professionals, a global organization with a mission to provide wheelchair users worldwide with the best technology and service [10].

Occupational therapists (OTs) are recognized as professionals for whom “assistive technology provision is a core competency” and thus should receive adequate training to meet the mobility needs of people with disabilities through adequate provision [11]. However, global evidence suggests that wheelchair-related content currently taught is not consistent and may be limited, both in high-income [12, 13] and LMIC countries alike [14, 15], albeit facing different challenges, despite development and dissemination of open-source capacity-building resources for wheelchair services [10, 16–18]. Nonetheless, some progress has been reported by rehabilitation training programs including occupational therapy (OT), physical therapy (PT), and prosthetics and orthotics (P&O) training in certain regions [19, 20]. In a recent study of wheelchair service provision content, offered in rehabilitation programs by 14 academic institutions across different nations (*N* = 11), Fung and collaborators identified strategies that help programs integrate the content into their curriculums [20]. Although the authors recognize the small sample size as a limitation, the study offers initial insight into the current status of wheelchair provision content across different geographical contexts [20]. Of the 14 nations included in this study, 8 corresponded to LMIC income settings [20]. Furthermore, the World Federation of Occupational Therapists (WFOT) recently published a report on their global survey for assistive technology provision across member organizations (i.e., occupational therapy national associations). With over 1020 individual therapist responses and 52 national associations' representatives, manual wheelchairs were identified as the second most common prescribed assistive technology product (59%) [21]. When grouped by the World Bank income categories, responses revealed that only 15% (*n* = 3) of member countries from LMIC reported access to assistive technology met the needs of their population well, compared to 84% (*n* = 26) in high-income nations [21].

Specific to the Colombian context, the congress ratified the United Nations Convention on the Rights of Persons with Disabilities and enacted the disability law in 2013 [22]. Although the enactment of this legislation is an indication of the country's commitment to access to rehabilitation and assistive technology [23], wheelchairs are not covered in the mandatory health plan [24]. In addition, little is known about the state of wheelchair education in the country, aside for a recent pilot study of two Physiotherapy programs [15, 24]. The Colombian Occupational Therapy Law, Law 949 of 2005, specifically addresses the important role of occupational therapists in the provision of assistive technology in the country [25]. Article 22 states “the occupational therapist will, autonomously, prescribe, design, elaborate, or adapt appropriate assistive technology equipment required by services users” [25]. In addition, the law specifically bans occupational therapists from delegating these functions to personnel trained at other levels such as technicians, technologists, and others [25]. Given the role of occupational therapists in-country, the purpose of this descriptive study was to evaluate the current state of basic manual wheelchair provision knowledge in final year entry-level OT programs in Colombia (i.e., undergraduate) as a first step to understand in-country educational needs and inform the development of culturally relevant strategies.

## 2. Materials and Methods

The Ethics Committee at Universidad CES (Colombia) approved this study in its August 2018 session. Participating programs (described below) provided consent for participation, agreeing to send the study information and test links to individual students. Individual students who agreed to participate provided implied consent. No personal health information was collected.

### 2.1. Recruitment

As of July 2019, Colombia had 11 active undergraduate OT programs that ranged in total duration from 8 to 10 semesters [26]. Inclusion criteria for participating programs were (1) an entry-level occupational therapy program with registration and approval from the Colombian Ministry of Education; (2) a program with at least 1 student in the final year of studies according to the program's duration. Students in the final year were chosen as the intent was to measure knowledge closer to the entry-to-practice point.

The authors contacted each program director to explain the purpose of the study and invite them to participate. One interesting program was excluded from participating because its most senior students were in 3^rd^ year (6^th^ semester) of a 4-year program at the time of the study and therefore did not meet the inclusion criteria. After the program director indicated an interest in participating, an email with the informed consent template in the body of the email, a brief motivational video explaining the importance of participating in the study, and instructions for the International Society of Wheelchair Professionals (ISWP) Wheelchair Service Provision Basic Knowledge Test (written and video demonstration) were sent. The director was asked to forward the email to the final year students in the program. All the communications were in Spanish.

### 2.2. Outcome Measure

ISWP offers the Wheelchair Service Provision Basic Knowledge Test [27, 28]. The test is aligned with the WHO Wheelchair Guidelines' definition of the “basic level” as the one required to meet the needs of users that are able to sit upright without additional postural support [1]. The test assesses 7 domain scores, based on the 8 steps recommended by WHO as necessary for appropriate manual wheelchair provision with the exclusion of step 1: referral and appointment [1, 28]:

1. Assessment: In this step, trained personnel work with the user to identify the individual needs while considering the environmental and individual conditions that impact them. The outcome of this step is to identify an appropriate wheelchair, determine the need for postural support and the training needs
2. Prescription: Based on the information gathered during the assessment, prescription involves the selection of a wheelchair and its specifications
3. Production: Refers to the assembly and safety of the prescribed wheelchair
4. Fitting: Includes the adjustment of the wheelchair to match the characteristics of the user
5. User training: This domain refers to the methods that can best help the user perform transfers and mobility activities with the use of the wheelchair
6. Follow-up and maintenance: Includes the repair, maintenance needs, and follow-up activities appropriate for the user needs and the wheelchair characteristics
7. Process: This domain assesses personnel's understanding of the continuum of provision from referral to funding and ordering [1, 28].

In addition to specific domain scores, a total score is provided. The reference passing threshold score is 70% [28]. The ISWP Basic Knowledge Test was developed by an Assessment Development Task Force through 6 steps: domain selection, question development, alpha testing, beta testing, pilot testing, and evaluation [28]. The test was translated into Spanish by two bilingual subject matter experts and then taken by 30 people who had the opportunity to comment on the relevance and clarity of the questions and responses they received. ISWP is currently leading a committee that is conducting an assessment of the Test's performance at the item (difficulty and discrimination index) and domain (reliability) levels. The results of this analysis will be published soon.

To further explain the context in which this study was conducted, we identified for each participating program the courses in the study plan with a name explicitly related to assistive technology.

### 2.3. Data Analysis

ISWP test results include individual domain and total scores. The authors grouped individual students' responses by the university and the universities were labelled from A to G to protect the confidentiality of participating programs and their individual students. The authors used descriptive statistics (mean and standard deviation) to summarize the data, and a parametric one-sample *t* test to determine whether the difference between the sample total score mean differs systematically from the passing threshold value.

## 3. Results

**Figure 1:**
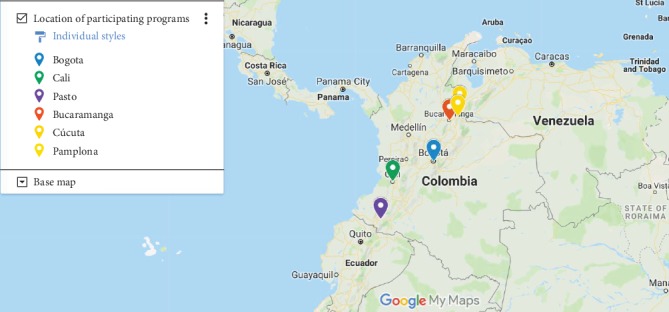
Geographical location of participating programs.

A total of 83 final year OT students from 7 universities in Colombia took the ISWP Wheelchair Service Provision Basic Knowledge Test in Spanish. Three universities did not participate in the study even though they indicated an initial interest in doing so. However, protests in public educational institutions occurring during the time frame of this study prevented their participation.  Figure 1 illustrates the geographical location of the participating programs. Two of the participating programs were located in Bogota (the country's capital city), one in Cali (the third-largest city in the country), one in Pasto, two in the state of Norte de Santander (Cucuta and Pamplona), and one in the state of Santander (Bucaramanga), located in the north-eastern region of the country. Two of the three programs that did not participate are in Bogota and one is located in Cali.  Table 1 presents the programs' courses related to assistive technology.  Table 2 includes the domain and total scores of each of the participating programs.

The total score of the sample was statistically significantly lower by 23.65% (95% CI, 21.34 to 25.97) than the passing threshold of 70%, *t*(82) = −20.32, *p* = .0005, *d* = 2.23. The latter reflects a large effect size, above two standard deviations.

## 4. Discussion and Conclusions

This study evaluated the current basic wheelchair provision knowledge of final year undergraduate occupational students in Colombia. The findings are concerning as none of the students obtained the minimum threshold (70%) to pass the test, and the mean total score of the sample was statistically significantly below the passing threshold. In addition, the difference was statistically large (*d* > .8). The assessment was the domain that had the better results while user training and fitting, the lowest. These results suggest that the current wheelchair provision education received in these OT programs do not meet the WHO guidelines on appropriate wheelchair provision. In Colombia, in addition to the OT law, OTs are recognized as critical stakeholders in the interdisciplinary team that leads the wheelchair provision in Bogota's AT guidelines, the Military Forces AT provision protocol, and health care guidelines recommendations for persons with disabilities [29–31].

A study that explored barriers and facilitators to including wheelchair service provision content in formal academic training reported that context setting affects the level of content taught [20]. Our findings support these studies. In Colombia, wheelchairs are not included in the mandatory health care plan and require a legal appeal to access them [24]. Therefore, the lack of a clear care pathway to access wheelchair products and services may be an explanation of the limited inclusion of wheelchair service content as suggested by the outcomes in this study.

The results may be used in conjunction with the previously reported knowledge of PT students, to raise the awareness on the need and priority for improved AT access to warrant the rights of people with mobility impairments and their participation in the community [32]. Strengthening the content taught in formal OT curricula must be paired with awareness and advocacy efforts, so the care path is established in the country, including the minimum personnel qualifications required to meet WHO guidelines. It is important that the national professional and academic OT organizations are involved [33] to guide the process that interprofessional will propose strategies to improve access to appropriate wheelchairs, including the information management system [34].

There is a need to promote coordinated and sustained actions to improve the current wheelchair provision content in Colombian programs. The immediate next step is to understand the current content taught (what, why, and how) to identify the opportunities for improvement, both in the specific assistive technology-related courses presented in Table 1 as well as in other courses and practicums. The following step will include to build the instructors' knowledge and skills. Instructors may participate in local or regional continuing education opportunities such as the Colombian Seating Symposium or the Latin American Seating Symposium, respectively. Online up-to-date opportunities include those offered by the International Society of Wheelchair Professionals and the Massive Online Open Course (MOOC) offered by Physiopedia in English and French [35]. In addition, the university professors could base the strengthening of the content and learning activities, on the Seating and Mobility Academic Resource Toolkit (SMART; http://smart.wheelchairnetwork.org/) and its Academic Training Partners community offered by the ISWP which has some resources in Spanish [10, 36, 37]. Instructors and programs can consider integrating the new content using blended methodologies (online + in − person), which have been proven to have an increase in knowledge for manual wheelchair training [17, 18, 38]. Last, other resources freely available in Spanish include the WHO Wheelchair Service Training Package series that includes a Training of Trainers package with useful teaching methodologies [39–43]. A mentoring/support system may be established between the instructors that lead the wheelchair-related content. An example of a working strategy is in Japan, in which OTs have an “AT advice system” where they can have guidance on what best AT matches the user's needs. The system is led by mentors that are specialized in AT [44]. In Canada, a community of practice among OT programs has been established to improve the education of Canadian OTs in the area of wheelchair provision according to WHO guidelines [45].

Other resources are available that complement the WHO proposed content and that may be relevant to the current known needs of the context for each domain. In the assessment domain, the WHO (2014) launched the Training Package for Wheelchair service-basic level in Spanish [46]. This package is free and available online and includes the theoretical and practical foundations for the individual assessment of wheelchair users who are able to maintain the seating position independently, with no additional postural support required. Given that students in our sample exhibited the highest level of performance in this domain (57.6%), this refresher may provide a structured approach to assessment that may reinforce such skills. The information contained in this package can also provide additional training resources for OT students in the prescription domain, as it contains information relevant to this area. Furthermore, the Health Secretary in Bogotá (Colombia's capital city) published guidelines for AT provision that includes a protocol for wheelchair prescription [29]. In the process domain, curricular innovations should be directed towards increasing awareness of regulation and service pathways available in the local context, including legal, infrastructure, and funding. Similarly, students could benefit from curricular content that offers links with vendors available in the local context, as well as the WHO guidelines for wheelchair prescription which includes a dedicated section on recommendations for how to design, evaluate, and select wheelchairs. The fitting domain is particularly concerning given the low performance of Colombian OT students in this domain (23,7%), lower than any other domain included in the test. This domain also poses an additional challenge in that like assessment, training would be enhanced by in-person workshops within the curriculum. As such, the training of educators is a key strategy to overcome this challenge. Moreover, other organizations have developed specific resources around fitting that are freely available online and might supplement the learning of OT students in this domain. For example, the Academy of Neurologic Physical Therapy has developed a free online resource directed at Wheelchair Fitting and Measurement [47]. However, translation of this resource would be necessary to enhance uptake. Specific to the user training domain, wheelchair skills training has been reported in Colombia as an identified strategy needed by wheelchair users to improve their mobility and navigation through widespread inaccessible built environments [48]. There are structured manual and power wheelchair skills training programs, proven to increase occupational therapy students' skills whether the education intervention is distributed via university courses or through a condensed practice boot-camp approach [49–51]. Evaluation of students' wheelchair skills learning is also possible with the use of different measures developed to accompany the training program, such as a questionnaire or newly developed self-efficacy assessment [52]. A cross-cultural translation and validation of the manual wheelchair skills questionnaire outcome measure is available in Colombia [53]. As such, these strategies provide promising advances that can increase competency for occupational therapy students specific to wheelchair skills training.

The authors believe that the outcomes of this study are a step in the right direction towards aligning OT education with current global trends. First, in their recent AT report, one of the three WFOT strategic recommendations states that “WFOT will advocate for the role of occupational therapists in the assistive technology process and their need for training and contextually relevant strategies and supports” [21]. As such, understanding the current status of AT education in OT will enable Colombian programs and others around the world who pursue similar diagnostic measures, to strengthen their graduates' competencies in this area. Second, the WHO is urging Member States, like Colombia to include rehabilitation and assistive technology in their plans for universal health coverage as per its Rehabilitation 2030 and the Global Cooperation on Assistive Technology (GATE) initiatives [54, 55]. To increase access to rehabilitation and AT in healthcare systems, a systemic approach has to be undertaken that includes the 6 building blocks: leadership and governance, finance, workforce, service provision, essential medicines and AT, and information systems [56]. In this sense, our work contributes to the workforce building block and provides direction to Colombian programs and others who may choose to replicate this process, to advance in alignment with these global directives. Furthermore, the forthcoming WHO Rehabilitation Competency Framework [57] and the Global Report on Assistive Technology [58] must be used to inform the next steps. For example, these documents will be crucial when deciding the wheelchair provision-related knowledge, skills, attitudes, and behaviours that new OT professionals should have. Understanding these global initiatives provides opportunities for support and international collaborations that can build capacity in-country, recognizing the unique skills and needs of the local professionals and users.

## Figures and Tables

**Table 1:** Mandatory assistive technology-related courses offered in each program study plan.

University	AT-related course (year)
A	Orthotics and adaptations (2)
B	Orthotic aids (6); assistive technology (4)
C	Splints and special equipment (2)
D	Splints and special equipment (2)
E	Technology and occupation (3)
F	Rehabilitation technology (2)
G	Orthopedics and orthotics (3)

Table generated by the authors from program study plans available online. Occupational Therapy programs in Colombia vary between 4 and 5 years in length.

**Table 2:** Total and domain scores per university.

University (# students)	Assessment		Prescription		Process		Production		Fitting		User training		Follow-up		Total score	
	Ave (%)	SD	Ave (%)	SD	Ave (%)	SD	Ave (%)	SD	Ave (%)	SD	Ave (%)	SD	Ave (%)	SD	Ave (%)	SD
A (24)	57.0	17.9	49.7	15.2	51,7	23.7	37.5	20.7	24.6	11.0	49.4	15.2	45.8	31.0	46.6	12.7
B (14)	47.0	19.2	43.5	13.5	45.7	26.2	41.5	14.6	20.7	12.7	35.7	14.5	37.5	23.5	39.6	11.
C (11)	57.4	11	53.8	10.8	44.5	20.2	47.3	25.7	30.9	12.2	52.7	17.0	52.3	28.4	49.7	9.7
D (17)	53.6	10.9	51.1	11.9	48.2	21.6	45.7	20.3	29.4	13.0	44.3	10.8	50.0	21.7	47.0	7.5
E (9)	56.4	28.5	48.3	22.0	35.7	20.7	42.2	34.9	28.9	22.9	42.4	24.7	44.4	33.4	48.7	9.4
F (7)	63.2	12.2	46.4	16.6	61.4	18.6	45.7	29.9	21.4	14.6	42.9	12.7	39.3	31.8	47.4	5.7
G (1)	68.4	NA	66.7	NA	70.0	NA	80.0	NA	10.0	NA	46.7	NA	75.0	NA	57.3	NA
Total across domains	57.6		51.3		51.0		48.5		23.7		44.9		49.2		48.0	

## Data Availability

The domain and total scores data used to support the findings of this study are available from the corresponding author upon request.
